# Progress in Research on White Organic Light-Emitting Diodes Based on Ultrathin Emitting Layers

**DOI:** 10.3390/mi15050626

**Published:** 2024-05-07

**Authors:** Wencheng Zhao, Xiaolin Hu, Fankang Kong, Jihua Tang, Duxv Yan, Jintao Wang, Yuru Liu, Yuanping Sun, Ren Sheng, Ping Chen

**Affiliations:** 1 Institute of Physics and Electronic Information, Yantai University, Yantai 264005, China; zhaowench@yeah.net (W.Z.); xiaolinhu@s.ytu.edu.cn (X.H.); fankangkong@s.ytu.edu.cn (F.K.); 15251084233@s.ytu.edu.cn (J.T.); yandx316@s.ytu.edu.cn (D.Y.); ypsun@ytu.edu.cn (Y.S.); 2 Institute of Information Engineering, Yantai Institute of Technology, Yantai 264005, China; wangjintao@yitsd.edu.cn; 3 Institute of Engineering Training Center, Yantai University, Yantai 264005, China; lyr@ytu.edu.cn

**Keywords:** white organic light-emitting diode, ultrathin emitting layer, efficiency

## Abstract

White organic light-emitting diodes (WOLEDs) hold vast prospects in the fields of next-generation displays and solid-state lighting. Ultrathin emitting layers (UEMLs) have become a research hotspot because of their unique advantage. On the basis of simplifying the device structure and preparation process, they can achieve electroluminescent performance comparable to that of doped devices. In this review, we first discuss the working principles and advantages of WOLEDs based on UEML architecture, which can achieve low cost and more flexibility by simplifying the device structure and preparation process. Subsequently, the successful applications of doping and non-doping technologies in fluorescent, phosphorescent, and hybrid WOLEDs combined with UEMLs are discussed, and the operation mechanisms of these WOLEDs are emphasized briefly. We firmly believe that this article will bring new hope for the development of UEML-based WOLEDs in the future.

## 1. Introduction

Given that approximately 25% of global electricity consumption is allocated to illumination, which results in the emission of 190 million tons of carbon dioxide, organic light-emitting diodes (OLEDs) have emerged as promising technologies in solid-state lighting and flat-panel displays, owing to their unique advantages such as rapid response, wide viewing angles, sunlight-like characteristics, and flexibility [1,2,3,4]. To achieve white organic light-emitting diodes (WOLEDs), dyes with broad emission covering the visible spectrum are required. Hence, the fundamental principle for achieving white emission involves blending emitters of two complementary colors or three primary colors. In recent years, various structures have been explored to achieve white emission, including (i) employing a blend of different colored dyes in a single emitting layer (EML), (ii) vertically stacking multiple EMLs in a single device, and (iii) combining multiple monochromatic OLEDs either vertically (series-connected OLEDs) or horizontally (space-abled OLEDs). It has been 15 years since J. Kido and his team first reported WOLEDs [5]. While WOLEDs have surpassed incandescent lamps and even fluorescent lights in terms of emission efficiency, they are now entering mainstream display markets. Due to their high efficiency, lightweight design, and cost-effectiveness, WOLEDs are also being explored for next-generation lighting applications [6]. However, the reported WOLEDs always have much more complicated structures, which causes higher costs for commercial manufacturing. The key to preparing WOLEDs is to simplify the fabrication process while ensuring the accuracy and repeatability of the devices [7]. Therefore, researchers worldwide are working to gradually simplify the device fabrication process while ensuring the performance of WOLEDs.

Over the past three decades, it has been firmly established that a well-designed EML is crucial for achieving high performance in WOLEDs [8]. Among these, introducing an ultrathin emitting layer (UEML) is a promising and innovative approach that dramatically simplifies device fabrication and reduces device costs. Notably, the UEML structure presents an advantageous alternative to traditional doping techniques while retaining their doping effects [9]. Experimental results indicate that precise control of the UEML thickness can be achieved by monitoring a calibrated quartz crystal sensor connected to a quartz crystal vibration probe outside the vacuum chamber. Due to the intrinsic properties of sub-monolayers, introducing a UEML with a thickness of less than 1 nm does not significantly impact the charge carrier behavior of the device [10]. Furthermore, a UEML represents a particular doping emitter without conventional doping processes, making it a potential replacement for traditionally doped EMLs. Compared to conventional host-guest doping systems, devices based on UEMLs offer several advantages. Firstly, there is no need to consider the alignment of energy levels between the host and dopant. Secondly, UEML-based devices can emit relatively independently. Thirdly, the UEML approach can result in at least 70% material cost savings. In addition, the UEML simplifies the device process on the basis of the complete utilization of excitons, so a UEML-based WOLED design is more flexible and environmentally friendly. Presently, UEML-based highly efficient WOLEDs have been widely reported, achieving external quantum efficiency (EQE) exceeding 20% [11]. Multiple UEMLs are often introduced in devices to achieve WOLEDs. The incorporation of multi-UEMLs increases the number of emission sites, improving the utilization of excitons.

In this review, we place emphasis on the research progress of WOLEDs utilizing UEMLs. First, the working mechanism and distinctive advantages of WOLEDs based on UEMLs are discussed. Second, the design strategies and applications of WOLEDs that combine UEMLs with doped layers, including fluorescent, phosphorescent, and hybrid WOLEDs, are summarized. Finally, the successful applications and the operation mechanisms of doping-free technology in fluorescent, phosphorescent, and hybrid WOLEDs combined with UEMLs are emphasized. Abbreviation section lists the full spellings of all terms referenced in this review.

## 2. Working Mechanism of WOLEDs Based on UEMLs

**Figure 1:**
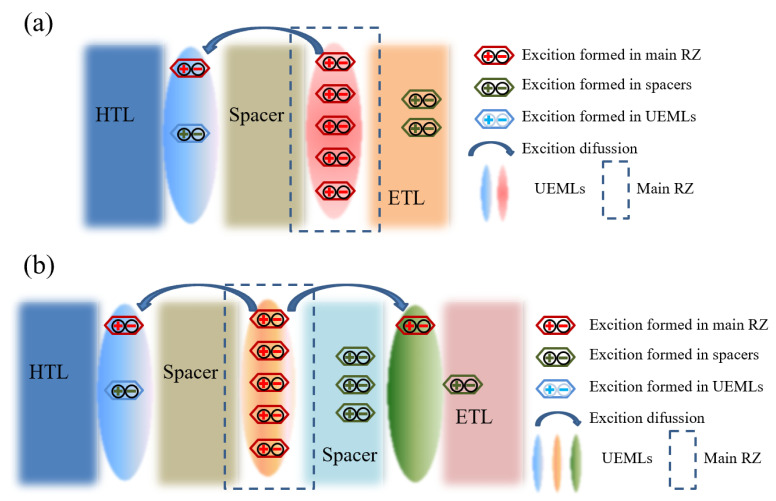
Possible working mechanism for (**a**) double color UEML-based WOLEDs; (**b**) triplet color UEML-based WOLEDs.

The realization of WOLED involves blending three primary colors (red, green, and blue) in specific proportions to achieve collective white light emission [12]. Due to the broad spectral coverages of emission materials, achieving white light emissions is also feasible by combining complementary colors like yellow and blue. In order to simplify the device structure while achieving high efficiency and spectrally stable white light emission, various doped and non-doped fluorescent, phosphorescent, and hybrid white light devices based on the structure of UEMLs have been developed. For WOLEDs based on UEMLs, the exciton diffusion and energy transfer are shown in Figure 1. UEMLs offer unique advantages in cost reduction and simplifying device structures. The selection of materials and the rationality of device structures play crucial roles in the performance of WOLEDs based on UEMLs [13]. Considering that the main mechanism of WOLEDs based on UEMLs is typically energy transfer between host and guest molecules, the efficiency of devices can be enhanced by effectively managing excitons through rational adjustments in the thickness and positioning of UEMLs, thereby broadening the exciton recombination zone and reducing triplet–triplet annihilation (TTA) [14]. For device stability, continual innovation in multifunctional emitting materials and rational device structure design is essential [15].

**Figure 2:**
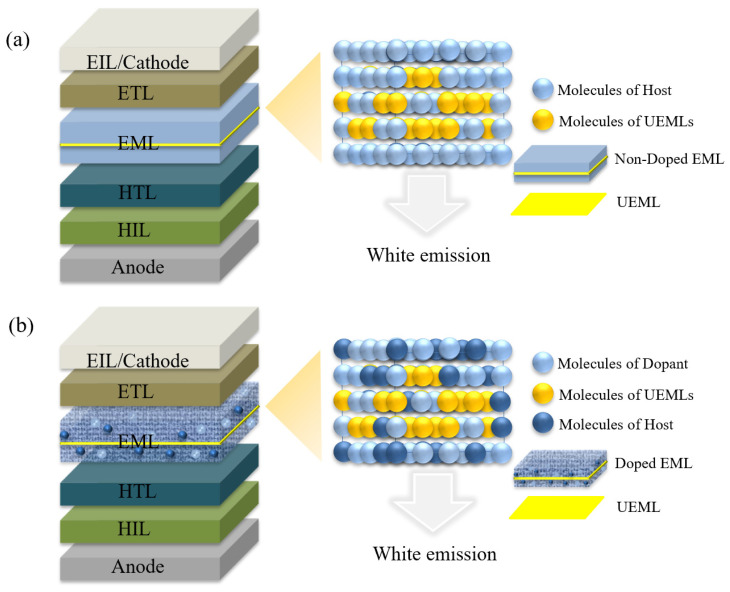
Schematic device structure for UEML-based WOLEDs with (**a**) non-doped layer and (**b**) doped layer.

The most common technique for preparing doped layers is co-evaporation under high vacuum to change the doping concentration of the host and guest by controlling the deposition rate. On the one hand, the preparation of doped EMLs requires rational consideration of energy level matching between different materials. On the other hand, precise control of the doping concentration is needed. The UEML is a very thin layer of organic material used to produce white light, usually less than 1 nm [16]. According to atomic force microscopy (AFM) images, the molecules in the UEML occupy the concave–convex positions of the underlying surface like islands [17]. There are many ways to prepare and deposit UEMLs. For example, the solution method can be used to prepare UEMLs by dissolving the organic material in a suitable solvent and coating the solution on the substrate by spraying, dipping, or printing [18]. However, according to the characteristics of organic materials, in order to make the UEML more uniform, the deposition technology of organic molecules is usually used [19]. The introduction of UEMLs does not significantly affect the behavior of the carriers, while the doping effect can be achieved without the use of doping techniques, as shown in Figure 2.

UEML-based full-fluorescent WOLEDs possess distinctive cost advantages [20]. Fluorescent materials utilize the first excited singlet state (S1) to achieve fluorescence emission. Fluorescent materials exhibit good material stabilities and cost advantages. However, the internal quantum efficiency (IQE) of fluorescent materials is limited to 25% at most, and the remaining 75% is converted into thermal energy through processes such as vibration [21]. Due to limitations imposed by the emission mechanism of the materials themselves, the efficiencies of full-fluorescent WOLEDs are generally low.

In the process of emission, phosphorescent materials can fully utilize the energy between singlet and triplet excitons (T1), theoretically achieving a 100% IQE [22], which significantly enhances the EQE of WOLEDs. Despite the excellent performance of phosphorescent materials, their limited heavy metal reserves, high prices, and environmental pollution are challenges. UEMLs can offer flexibility in design for optimal device performance, which reduces the use of expensive phosphorescent materials and simplifies device structures [23]. In general, to ensure intense blue light emission, UEML-based phosphorescent WOLEDs are usually realized by combining a blue doping layer with a complementary color UEML.

To overcome the short lifetime of blue phosphorescent materials, one approach is to combining long-lifetime blue fluorophors with highly complementary phosphorescent UEMLs to prepare hybrid WOLEDs, thereby improving their lifespan and reducing manufacturing costs [24]. By inserting phosphorescent UEMLs into blue fluorescent EMLs, it is possible to develop highly efficient fluorescent/phosphorescent hybrid WOLEDs [25]. In order to achieve the efficient utilization of triplet excitons in fluorescent materials, efficient Dexter energy transfer (DET) is necessary from blue fluorescent EML to complementary EML, which is a short-range action process with a typical ratio of 1 nm [26]. On the other hand, it is necessary to regulate the Förster energy transfer (*FRET*) rate between fluorescent and phosphorescent layers to reach sufficient blue emission, which can be expressed as follows [27]:

(1) $$k_{FRET}=\frac{1}{\tau_{p}}{\left(\frac{R_{0}}{R_{DA}}\right)}^{6}$$

where $\tau_{p}$ is the intrinsic radiative decay lifetime of the hosts in the absence of guests, $R_{0}$ is the Förster radius, and $R_{DA}$ is the host-to-guest distance. Therefore, $R_{DA}$ between the blue layer and complementary UEML should be controlled reasonably, increasing complexity in the device structure design.

In order to overcome the bottleneck of fluorescent and phosphorescent materials, thermally activated delayed fluorescence (TADF) materials are proposed to replace traditional blue fluorescent materials in hybrid WOLEDs, which can achieve the reverse intersystem crossing (*RISC*) process due to the small energy gap ($\Delta E_{ST}$) between S1 and T1 and result in theoretical IQE of 100% [28]. TADF materials typically adopt a donor–acceptor structure with a high twist angle to minimize $\Delta E_{ST}$, thus possessing strong intermolecular charge transfer characteristics [29]. This property broadens the emission spectrum full width at half maxima (FWHM), offering certain advantages in the preparation of WOLEDs. Substituting traditional fluorescent materials with TADF materials exploits their efficient *RISC* capability, suppressing non-radiative transitions of triplet excitons so that *FRET* is facilitated from short-wave emitters to long-wave emitters, significantly improving the utilization of triplet excitons [30].

The exciplex formed between the electron-donor and electron-acceptor also possesses an efficient *RISC* process due to a small $\Delta E_{ST}$, enabling the triplet excitons to undergo an upconversion to singlet excitons by absorbing thermal energy from the environment [31]. The delayed fluorescence generation is consistent with the mechanism observed in TADF materials, known as E-type delayed fluorescence. The rate expression for the *RISC* process in an exciplex is as follows [32]:

(2) $$k_{RISC}=A\exp\left(-\frac{\Delta E_{ST}}{k_{B}T}\right)$$

where $k_{RISC}$ represents the rate of the *RISC* process, A is the pre-exponential factor, $k_{B}$ is the Boltzmann constant, and *T* is the temperature. It is evident that the *RISC* rate in an exciplex is dependent on both $\Delta E_{ST}$ and *T*, where higher temperatures and smaller $\Delta E_{ST}$ values will increase the *RISC* process rate. Given that environmental temperature is challenging to alter in most situations, the design of an exciplex with a smaller $\Delta E_{ST}$ becomes crucial. The unique properties of an exciplex are widespread applications in the emission of WOLEDs. On the one hand, an exciplex can serve as a blue emitter and combine with UEMLs to achieve WOLEDs [33]. On the other hand, an exciplex can also serve as the host in the UEMLs, participating the carrier transport and energy transfer within the device [34].

## 3. Doping WOLEDs with UEMLs

Generally, white emission in OLEDs can be achieved by mixing complementary colors, such as blue and orange, or primary colors, such as red, green, and blue, emitted from single or multiple EMLs [35]. The traditional method for producing WOLEDs involves co-evaporating two or more dyes. While complete doping techniques can enhance energy transfer and improve electroluminescence (EL) efficiency, manufacturing is burdensome. Therefore, incorporating one or more materials in the form of UEMLs into the doping device not only enables the production of WOLEDs but also dramatically reduces the complexity of the fabrication process.

### 3.1. Fluorescent Doping WOLEDs with UEMLs

Fluorescent doping WOLEDs with UEMLs has great advantages in terms of stability and material cost. Early efforts have promoted the development of WOLEDs based on UEMLs. Currently, UEML-based fluorescent WOLEDs without exciplex have been extensively studied [29].

P-bis(p-N, N-diphenylaminostyryl)benzene (DSA-ph) is one of the most efficient blue dopants and is widely used in the production of high-efficiency blue and white devices [36,37]. In 2011, Xue et al. prepared double-color WOLEDs consisting of a blue EML doped with DSA-ph in 2-methyl-9,10-di(2-naphthyl)anthracene (MADN) host and a UEML of 5,6,11,12-tetraphenyl-naphthacene (Rubrene) as the yellow-EML. The thickness of the Rubrene significantly affected the performance of the device, with experimental results showing severe quenching at 2 nm and 3 nm and higher EL efficiency at 1 nm. Bright white light of over 10,000 cd/m² was successfully achieved at a low driving voltage of 8 V. The maximum EL efficiency and power efficiency (PE) were 8.69 cd/A at 7 V and 5.5 Lm/W at 4 V [38].

**Figure 3:**
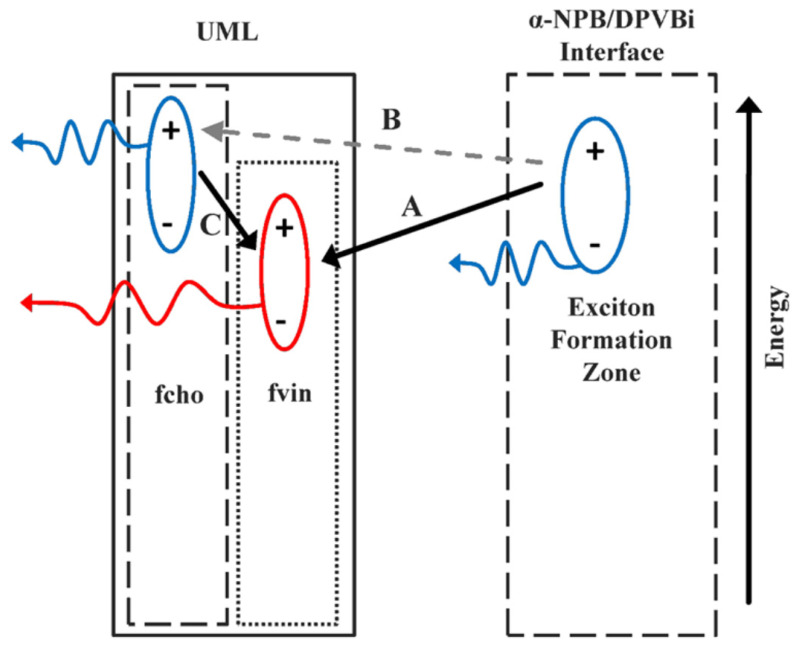
Schematic emission mechanisms of OLED within the UPL film [41].

To improve process simplicity and repeatability, a single emitting white layer of different compounds can be “pre-mixed” in one evaporation boat [39,40]. The advantages of UEMLs, such as low current dependence on color coordinates and fine control of chromaticity, combined with the reproducibility and fine control of pre-mixed materials and extremely low doping rates, can mitigate the issue of quenching in fluorescent materials. In 2013, T. Jeon et al. proposed a method for achieving precise color control in WOLEDs based on ultrathin premixed layer (UPL), as shown in the Figure 3. The UPL consisted of two molecules with similar structures and melting temperatures, the red emission of 4-di(4′-tert-butylbiphenyl-4-yl)aminobiphenyl-4′-yl)acrylonitrile (fvin) and the green emission of 4-di(4′-tert-butylbiphenyl-4-yl)aminobenzaldehyde (fcho), which mixed in one evaporation boat to produce a film with the same and reproducible composition as the premixture. By evaporating this blend in a 1 nm thick layer in the hole transport layer (HTL) α-NPB of a standard fluorescent OLED structure, combined with the blue emission from the heterojunction, white light with the Commission Internationale de l’Eclairage (CIE) of (0.34, 0.34) and color rendering index (CRI) of 72 was obtained with good color stability under injected current [41].

### 3.2. Phosphorescent Doping WOLEDs with UEMLs

When using the UEML method to prepare WOLEDs, ultrathin dye with a thickness of less than 0.5 nm may only be dispersed on the surface of the underlying organic layer, leaving many unoccupied positions on the surface. When the second UEML is deposited on the surface of the first layer without any interlayer (IL), the second ultrathin dye may act as a gap dopant, randomly filling the unoccupied positions. Based on this concept, researchers have developed IL-free phosphorescent doping WOLEDs with UEMLs [31]. The color balance of the device can be actively adjusted in a more straightforward and controllable manner by varying the thickness of the UEMLs, and the IL-free structure reduces the number of interfaces, significantly improving the efficiency of devices.

In common co-doped host systems, the dopant typically plays a role in trapping charge carriers, which inevitably increases the operating voltage [42]. In contrast to common co-doped host systems, a highly efficient WOLED can be constructed using the delta-doping method by introducing the undoped UEML into the EML. The emission color of WOLEDs with a delta-doping EML usually depends on the position and thickness of the delta-doping layer [43,44]. In 2011, Ma et al. achieved both high efficiency and improved carrier injection by placing an undoped orange UEML at the interface of the HTL and electron transport layer (ETL), based on the concept of delta-doped EML. The experimental results showed that by using a more efficient phosphorescent dye, a WOLED with IQE close to 100% can be achieved through the delta-doping method. The WOLED exhibited very good warm white emission at different luminances with CIE coordinates of approximately (0.45, 0.45). At luminance of 1000 cd/m^2^, the EQE of the four-color WOLED was 15.5% with CRI of 87 [45]. Using this method, significant improvement in spectral and efficiency was achieved, but there was no change in electrical performance.

Multi-EMLs structures are often composed of two or three doped layers in different multi-EMLs, which receive special attention due to their relative ease in controlling doping concentrations and producing high-efficiency WOLEDs [46,47]. Factors that affect the color stability of WOLEDs with multi-EMLs include carrier trapping, exciton quenching, and TTA [48]. To match different dyes in different EMLs, the host in each EML is usually different. In 2014, Zhu et al. achieved high PE in bilayer all-phosphorescent WOLEDs by inserting undoped orange UEML of bis(2-phenyl-benzothiozolato-N,C^2’^)iridium(acetylacetonate) [Ir(bt)_2_(acac)] between two doped blue EMLs. The double blue layers were composed of 10% bis(3,5-difluoro-2–(2-pyridyl)phenyl-(2-carboxypyridyl))iridium III (FIrpic) doped 4,4,4″-tris(N-carbazolyl)triphenyl-amine (TCTA) and 20% FIrpic doped 2,6-bis(3-(carbazol-9-yl)phenyl)-pyridine (26DCzPPy). The device exhibited maximum PE of 63.2 Lm/W, current efficiency (CE) of 59.3 cd/A, and EQE of 23.1%, which slightly decreased to 53.4 Lm/W, 57.1 cd/A, and 22.2% at 1000 cd/m^2^ [49]. This excellent performance fully demonstrated the advantage of UEMLs in constructing high-efficiency WOLEDs.

Xu et al. studied the multi-EMLs structure for phosphorescent WOLEDs in 2016. This structure consisted of two independent blue layers and a red–green co-doped UEML sandwiched between them. The hole transporting material (HTM) of TCTA and bipolar transporting material of DCzPPy were chosen as the two host materials for blue emitter FIrpic. In between the two blue EMLs, red emitter bis(2-phenylquinoline)-(acetylacetonate)-iridium(III) [Ir(_2_-phq)_2_(acac)] and green emitter fac-tris(2-phenyl-pyridinato)-iridium(III) [Ir(ppy)_3_] were doped into an ultrathin TCTA layer. By carefully selecting the doping components and optimizing the layer thickness, the WOLED achieved EQE close to 20% and high PE exceeding 40 Lm/W at 1000 cd/m^2^ with low-efficiency roll-off and minimal color shift [50]. Through systematic investigation of various energy transfer pathways between hosts and dopants, they found that the co-doped UEMLs and two blue EMLs played a crucial role in achieving high efficiency and controllable white emission in the device [51].

**Figure 4:**
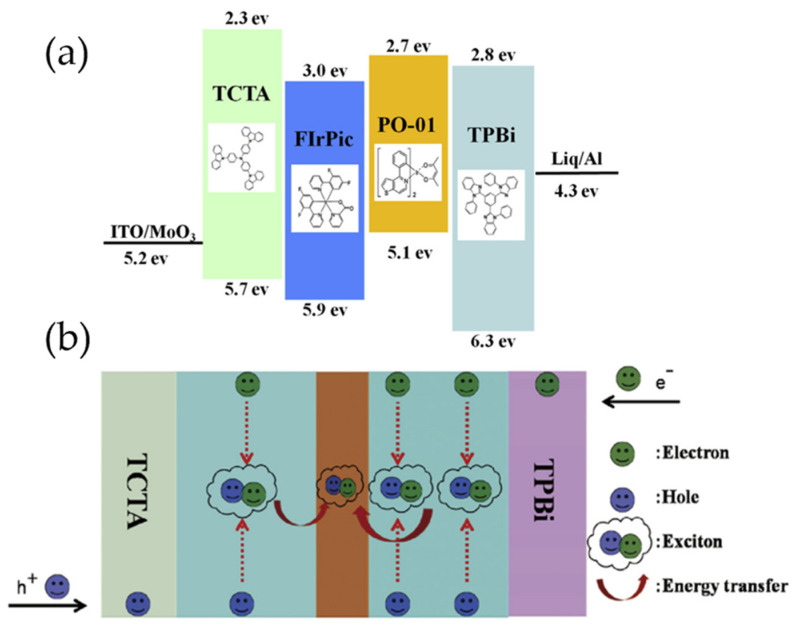
(**a**) The detailed energy level diagram and chemical structures of the materials; (**b**) the operational mechanism of white devices [55].

In traditional structures, all UEMLs are located at the interface with the highest expected concentrations of excitons, which can result in severe interface accumulation and exciton annihilation [23,52]. Therefore, the use of UEMLs in WOLEDs at high brightness levels poses a significant challenge due to efficiency roll-off issues [53]. In order to address this issue, our research group proposes the concept of double mixed-host EMLs, which involves the use of a mixed-host structure in the EML to achieve an appropriate width for the exciton recombination zone (RZ) and a balanced charge carrier transport [54]. By controlling the concentrations of HTM and electron transport material (ETM) in the mixed host, it is possible to reduce the exciton density on the EML and adjust the transport capabilities of charge carriers, thereby mitigating TTA and reducing efficiency roll-off. In 2016, our research team successfully demonstrated a high-efficiency white phosphorescent OLED device by combining double-blue-mixed-EMLs with a single orange UEML of Iridium(III) bis(4-phenylthieno[3,2-c]pyridinato-N, C2′) acetylacetonate (PO-01). TCTA with high hole-transport mobility was used as HTL, and 2,2′,2″-(1,3,5-Benzinetriyl)-tris(1-phenyl-1-H-benzimidazole) (TPBi) with high electron-transport mobility was used as ETL, which were blended as the mixed-host for the blue phosphorescent emitter of FIrPic. The mixed host EML had a ratio of 2:1 for the mixture of HTM and ETM near HTL and a ratio of 2:1 near ETL, as shown in Figure 4. This WOLED exhibited high efficiency of 40.8 Lm/W and 39.8 cd/A within wide luminance range, as well as low turn-on voltage of 2.71 V [55], owing to the further reduction of the interface energy barrier at the EML interface, which effectively promoted charge carrier transport and enlarged the exciton RZ [56]. Additionally, the change in CIE coordinates of the device was negligible (0.005, 0.008), indicating a stable emission spectrum, which could be attributed to three factors: the good bipolar conductivity of the mixed host layer, the use of UEMLs to mitigate the direct charge capture effect that would have caused color variation, and the rational arrangement of colors [57]. In the same year, our team successfully simplified the device structure and achieved high-efficiency phosphorescent WOLEDs by combining a single undoped UEML with a doped EML. The blue EML consisted of a mixed host structure of HTM (TCTA) and ETM (TPBi). The main feature of this device was the use of a blue mixed host and an orange UEML, resulting in broader RZ and more balanced charge carriers. The white device exhibited peak efficiency of 33.8 Lm/W and 32.2 cd/A with a slight efficiency roll-off. When the luminance was increased from 800 cd/m^2^ to 5000 cd/m^2^, the total CIE change was only (0.024, 0.005), which could be considered negligible, indicating that the white device showed a relatively stable emission spectrum due to the broader RZ in the EML [58]. Our series of experiments show that by not inserting any ILs or spacers between the two EMLs but by achieving a uniform distribution of excitons in the double-EMLs, it is possible to obtain devices with lower efficiency roll-off and a stable emission spectrum.

The research above has demonstrated that inserting a phosphorescent UEML between HTL and ETL can enhance the repeatability of the device. However, this device will inevitably experience significant efficiency roll-off at high brightness levels due to severe TTA processes [59]. Researchers achieve efficient WOLEDs by utilizing appropriate combinations of donors and acceptors, as well as a simplified structure for effective energy transfer from the exciplex to the EML. Due to the small singlet–triplet $\Delta E_{ST}$, 100% exciton utilization can theoretically be achieved through effective RISC from triplet to singlet [60,61]. The donor and acceptor of an exciplex are separated into two molecules. By selecting appropriate donor and acceptor materials, the energy levels of an exciplex can be easily adjusted. Therefore, WOLEDs based on an exciplex not only possess excellent bipolar carrier transport characteristics but also feature lower excited voltages compared with donor/acceptor materials, resulting in reduced operating voltage [62]. Proper management of excitons can also mitigate the negative impact of UEML. Thus, combining an exciplex with UEMLs is an effective method to simplify device manufacturing processes and improve exciton utilization.

In 2016, Wang et al. achieved the successful development of WOLED with low operating voltages, high efficiency, and minimal efficiency roll-off, accomplished by inserting an ultrathin, non-doped, highly efficient orange phosphorescent emitter layer of PO-01 within the blue layer of FIrpic. The exciplex consisted of N,N′-dicarbazolyl-3,5-benzene (mCP): bis-4,6-(3,5-di-3-pyridyl-phenyl)-2-methylpyrimidine (B3PYMPM) with a higher T1 (2.97 eV) than FIrpic as host. The device achieved maximum forward viewing efficiency of 64.5 cd/A, 75.3 Lm/W, and 20.0% without the use of light outcoupling techniques. At brightness of 1000 cd/m², it maintained 62.8 cd/A, 63.1 Lm/W, and 19.5% [63]. The device structure utilized wide-bandgap HTM and ETM with high T1, effectively confining excitons in the EML and achieving high efficiency. The reduction in driving voltage in the device was attributed to the significantly lower energy required for the excitation of the host compared to conventional hosts [64]. Additionally, the introduction of an orange UEML showed a negligible impact on charge transport characteristics, further contributing to achieving a low operating voltage.

**Figure 5:**
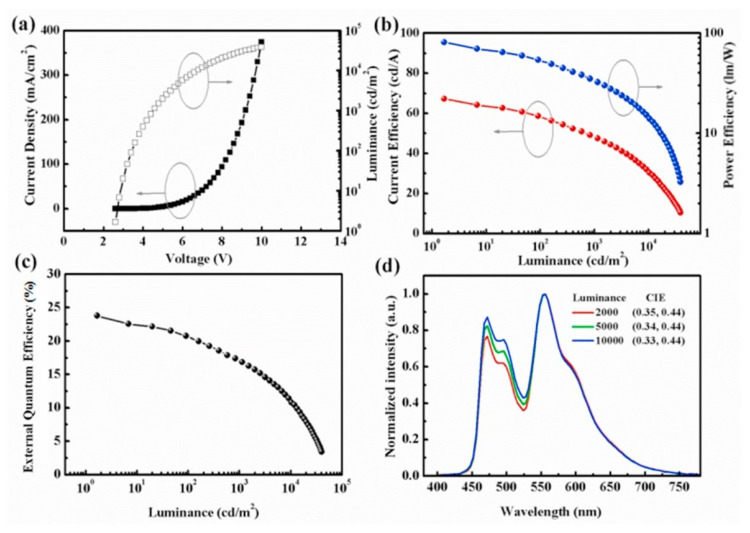
The performance of the resulting WOLEDs. (**a**) Current density–voltage–luminance; (**b**) current efficiency–luminance and power efficiency–luminance; (**c**) external quantum efficiency–luminance; (**d**) normalized intensity of the spectra [34].

**Figure 6:**
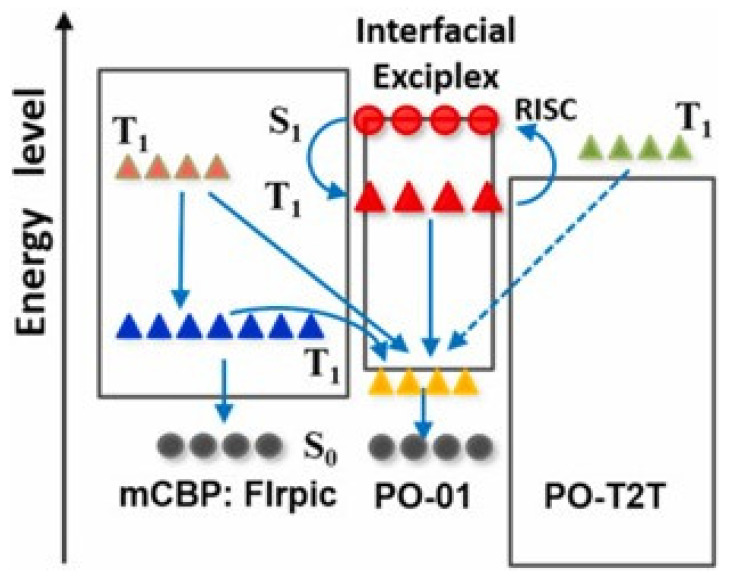
The exciton energy transfer mechanism from the exciton donors to the PO-01 in device [34].

In 2021, Zhang et al. achieved warm and cool phosphorescent WOLEDs based on interfacial exciplex co-hosts [65]. The device structure for the cool-state WOLED was ITO (180 nm)/HAT-CN (10 nm)/HAT-CN: TAPC (40 nm)/TAPC (5 nm)/4,4’-bis(3-mehtylcarbazol-9-yl)-2,2′-biphenyl (mCBP) (5 nm)/PO-01 (0.03 nm)/mCBP: FIrpic (15%, 2 nm)/1,3,5-triazine-2,4,6-triyl)tris(benzene3,1-diyl))tris(diphenylphosphine oxide (PO-T2T) (8 nm)/PO-T2T: Liq (10%, 30 nm)/Liq (1 nm)/Al (150 nm). The CIE coordinates were (0.26, 0.39), and the operating voltage for the cool-state device was 2.6 V. The maximum EQE, CE, and PE were 28.37%, 72.17 cd/A, and 87.17 Lm/W, respectively [34]. Furthermore, the warm-state WOLED was yielded by positioning PO-01 at the interface of mCBP: FIrpic /PO-T2T, achieving CIE coordinates of (0.35, 0.44) and low operating voltage of 2.6 V. The EQE was 23.80%, CE was 67.70 cd/A, and PE was 81.10 Lm/W [34]. Additionally, both devices exhibited stable color output in the brightness range of 2000 to 10,000 cd/m², as shown in Figure 5. The exciton energy transfer in the warm-state device is shown in Figure 6.

Research shows that the introduction of ILs can effectively solve the problem of energy barriers between different organic functional layers [66]. The performance of WOLEDs based on UEMLs is highly sensitive to the change of exciton RZ [67]. The exciton RZ and transport property between different EMLs can be reasonably managed using ILs, simplifying the regulation of device performance and improving the efficiency roll-off caused by exciton annihilation at high brightness levels.

In WOLEDs with UEMLs, the crucial factor for achieving high efficiency and CRI values lies in fine-tuning the thicknesses of the EML and IL [68]. The IL serves as a key component in facilitating efficient energy transfer between multi-EMLs [69,70]. The IL between multi-EMLs can achieve efficient energy transfer. In 2013, Yu et al. reported a high-efficiency and high-color-rendering phosphorescent WOLED with a structure of a red UEML–IL–green UEML, using a Pt complex as the blue phosphorescent emitter doped in mCP and an Ir complex as the green and red phosphorescent emitters doped in TPBi. By fine-tuning the thickness of the EML and the IL, high CRI of 80 with maximum CE of 29.2 cd/A and PE of 22.9 Lm/W without out-coupling enhancement were obtained. These values roll-off to 22.8 cd/A and 11.0 Lm/W at 1000 cd/m^2^ [71]. Compared with traditional devices, the research group found that the EL efficiency of the ultrathin device increased by about 2.0 times.

**Figure 7:**
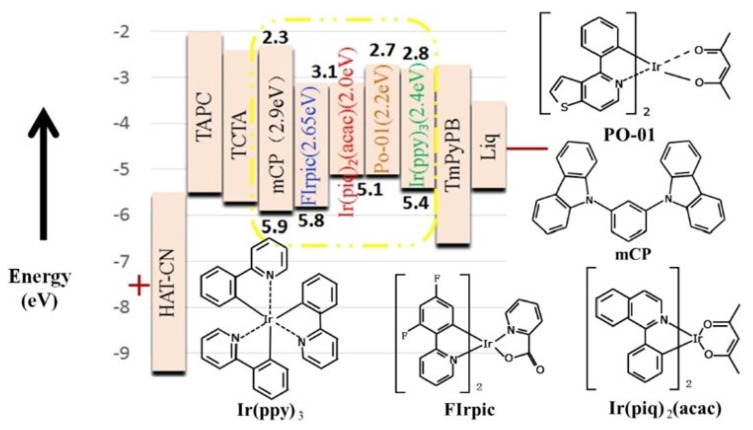
The schematic diagram of devices and the energy level/molecular structure of part materials [72].

In 2019, Wang et al. prepared a series of all-phosphorescent WOLEDs with three phosphorescent UEMLs (red, orange, green) separated by ILs (mCP) on both sides of the EML. The blue phosphorescent dye of FIrpic was doped in the bipolar host material of mCP with different doping concentrations. Since the triplet energy of the blue phosphorescent dye was higher than that of other phosphorescent dyes, energy transferred from both sides of the EML to the middle, as shown in Figure 7. The device reached maximum PE of 33.42 Lm/W and CRI of 85 with stable spectra from 5–8 V [72].

In contrast to a unipolar IL, mixed IL with bipolar transport promotes a uniform distribution of excitons throughout the entire EML. By altering the compositional ratio of the mixed bipolar IL, the spectral charge associated with applied voltage can be significantly reduced [73]. Additionally, adjusting the thickness of the bipolar IL allows the modulation of energy transfer from short wavelength emitter to long wavelength emitter, thereby tuning the emission color proportions to achieve coordinated white light emission [74]. Our research group proposes the combination of UEMLs with doped EMLs to achieve high-efficiency WOLEDs. This approach involves using an orange undoped UEML and a blue doped EML in WOLED. With optimum mixed ratio of 2:1 in the mixed IL composed of CBP and TPBi, a white device with PE of 40.1 Lm/W can be achieved, which is 2.1 times higher than that of devices using a CBP IL [75]. The mixed IL with interference energy levels prevents carrier accumulation at the CBP/TPBi interface, thereby improving PE by reducing the conduction voltage of the white device [76]. Additionally, the doping of TPBi effectively balances the charge of carrier transport in the emission region, which is another key factor in improving PE.

**Figure 8:**
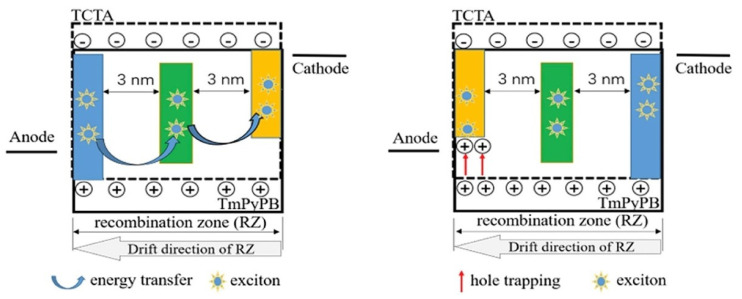
The work mechanism of spectral stability in WOLEDs with bipolar mixed spacer based on non-doped phosphorescent UEMLs [78].

In 2018, Yu et al. achieved high-efficiency phosphorescent WOLEDs using mixed bipolar ILs of TCTA: TmPyPB to separate undoped phosphorescent UEMLs. The CIE coordinates of both three-color and four-color WOLEDs show small variations with maximum efficiencies of 47.8 cd/A (48.8 Lm/W) and 44.9 cd/A (42.5 Lm/W), respectively [77]. By changing the thickness of PO-01, white light covering different times of the day with a similar solar radiation spectrum can be achieved. Moreover, the WOLED generally emits warm white light with correlated color temperature (CCT) ranging from 3686 K to 4700 K, showing potential for application in health lighting [77]. In 2019, Dai et al. prepared color-stable WOLEDs based on mixed bipolar ILs and non-doped phosphorescent UEMLs, demonstrating that the thickness and position of the UEML have a certain impact on the color stability of white light devices, as shown in Figure 8. The mixture of TCTA and TmPyPB served as the bipolar IL to regulate the distribution of carriers in the EML. The maximum CE, PE, and EQE of the device were 60.2 cd/A, 60 Lm/W, and 17.7%, respectively [78]. When the brightness increases from 1000 cd/m^2^ to 10,000 cd/m^2^, the obtained three-color white device showed insignificant color coordinate variations.

**Figure 9:**
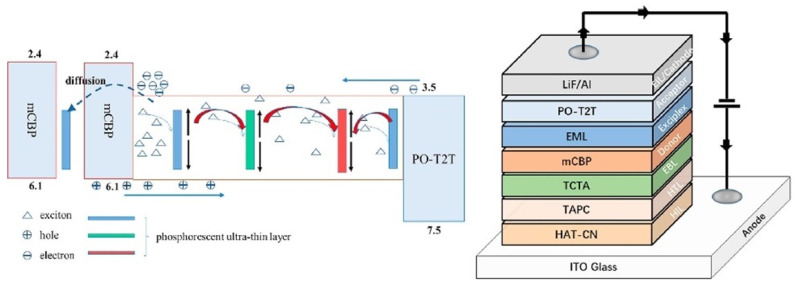
High efficiency and color quality WOLEDs have been demonstrated by strategically adjusting the positions of red, green, and blue phosphorescent UEMLs in an exciplex emitter and adding another channel to fully utilized CT state excitons [79].

In 2020, Zhang and their team successfully developed an efficient and high-color-rendering WOLED using an exciplex as the IL, as shown in Figure 9. The three-color phosphorescent UEMLs are inserted at different positions in the exciplex EML of mCBP: PO-T2T. The resulting WOLED exhibited maximum forward PE, CE, and EQE of 46.0 Lm/W, 36.4 cd/A, and 21.1%, respectively [79]. Additionally, the diffusion length of triplet excitons was exploited, which is typically an order of magnitude larger than that of singlet excitons (<10 nm) [43]. Based on the above concept, they optimized the blue light emission channel to optimize exciton utilization by inserting an FIrpic UEML with a thickness of 0.05 nm into the mCBP layer and separated from EML by 2 nm thick mCBP. The device with CRI exceeding 80 achieved maximum EQE, PE, and CE of 26.1%, 50 Lm/W, and 40 cd/A, respectively [79]. The high efficiency of the WOLED is attributed to the efficient energy transfer from exciplex ILs to FIrpic and barrier-free structure [80].

### 3.3. Hybrid Doping WOLEDs with UEMLs

The lack of efficient and stable blue phosphorescent emitters has hindered the development of phosphorescent OLEDs, resulting in poor lifetime performance [81]. Additionally, traps caused by TTA have also limited the progress of all-phosphorescent WOLEDs. Therefore, combining highly stable blue fluorescent emitters with efficient long-wavelength phosphorescent emitters, also known as hybrid WOLEDs, is a promising approach for achieving high efficiency and long lifetime [82]. These hybrid WOLEDs typically consist of a blue fluorescent emitter as the main emitter and several phosphorescent emitters emitting orange, green, or red light [83,84]. Since the groundbreaking discovery of a hybrid WOLED with maximum PE of 22.1 Lm/W by Forrest et al. in 2006, significant efforts have been made to improve the device performance of hybrid WOLEDs further [43].

Inserting a high triplet energy IL between the fluorescent and phosphorescent EMLs is a common method [85], which helps prevent unexpected exciton quenching from the phosphorescent to the fluorescent triplet state. This approach demonstrates the potential for achieving high-efficiency WOLEDs [86].

In addition to using doped emitters to construct efficient WOLEDs, the introduction of a single undoped dye layer (or undoped multi-UEMLs) in the EML has also been proposed. Hybrid WOLEDs using delta-doping technology have become a research hotspot due to their simple structure, good repeatability, suitability for low-cost applications, and potential for industrialization [81,83]. In 2013, Liu et al. first fabricated a new hybrid WOLED device composed of a blue UML using the delta-doping method. Through optimization, peak forward PE of 7.3 Lm/W and brightness of 46,923 cd/m^2^ were achieved [87]. The device efficiency roll-off was quite low, and the color was not very sensitive to the applied voltage. In addition, based on the introduction of an NPB IL, which can improve device performance, they proposed another new hybrid WOLED composed of double UEMLs (<1 nm). It is worth noting that the device showed low driving voltage (i.e., 2.7 V at 1 cd/m^2^, 3.4 V at 100 cd/m^2^, and 4.1 V at 1000 cd/m^2^), PE of 8.9 Lm/W, and CRI of 75. This achieves low driving voltage, high CRI (75), and high efficiency (8.9 Lm/W) [87].

**Figure 10:**
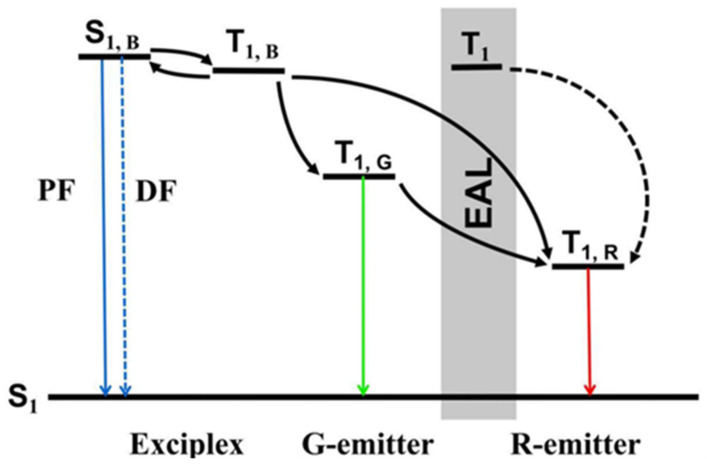
The function of EAL and energy transfer process in the device structures [90].

**Figure 11:**
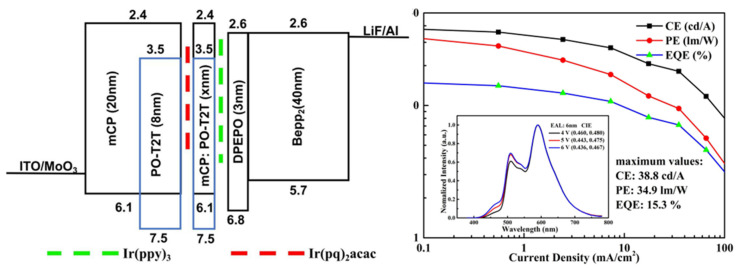
Diagram of the device structure with EAL [90].

Exciplex layers can serve as both hosts and emitters [88,89]. In 2018, Wang et al. inserted green and red phosphorescent UEMLs into an exciplex to achieve white emission. Here, mCP:PO-T2T served as a blue emitter and an excitons adjustment layer (EAL). DPEPO and Bepp_2_ have the same LUMO energy level (2.6 eV) and small energy barrier of 0.2 eV, ensuring efficient electron transport and injection. Furthermore, an exciplex acts as an EAL to control the exciton distribution and energy transfer between EMLs as shown in Figure 10. As shown in Figure 11, the maximum CE, PE, and EQE of the white light device reached 38.8 cd/A, 34.9 Lm/W, and 15.3%, respectively [90].

In 2012, Adachi et al. invented TADF emitters, in which the small $\Delta E_{ST}$ (<0.2 eV) between the S1 and T1 states allows for *RISC* from T1 to S1, providing an alternative method to achieve 100% IQE at room temperature without using expensive phosphorescent emitters [29]. TADF emitters also have a wider EL spectrum (FWHM of about 100 nm) compared to traditional fluorescent emitters, which is beneficial for achieving a high CRI for white light [91]. In 2021, our research group used a new HTM, N-([1,10-biphenyl]-2-yl)-N(9,9-dimethyl-9h-fluoren-2-yl)-9,90-spirobi[fluorene]-4-amine (FSF4A), and an ETM PO-T2T, to prepare a new exciplex host system. The improvement in efficiency is attributed to efficient energy transfer from the exciplex host to the phosphorescent dye. This device achieves ultra-low turn-on voltage of 2.2 V and maximum PE of 34.1 Lm/W with EQE of 12.4% [92].

In 2022, our research group reported WOLEDs employing a double *RISC* system combining blue-green TADF emitter and interface exciplex, which significantly reduces the concentrations of triplet excitons on the UEML and improves the EL performance of the device. At the same time, a 26DczPPy IL was introduced to suppress energy transfer between the blue-green TADF emitter and the orange UEML, enhancing the blue-green emission and achieving a double-color hybrid WOLED. The efficient utilization of excitons and suppression of exciton quenching are the main reasons for high performance and low-efficiency roll-off in the device [93]. The resulting device achieved maximum CE and PE of 52.4 cd/A and 60.9 Lm/W over a wide range of brightness with CIE changing to (0.002, 0.016) [22]. This is one of the best-performing hybrid WOLEDs based on UEMLs.

The aforementioned device structure, due to the introduction of additional ILs, can lead to an increase in driving voltage, as well as increased cost and complexity in the manufacturing process [56]. This results in less-than-ideal efficiency and higher production costs for hybrid WOLEDs. However, efforts have been made to simplify the device structure and eliminate doping processes, shorten the manufacturing process, and improve reproducibility [94]. Furthermore, the use of UEMLs is shown to effectively reduce costs while achieving high efficiency, ultra-high color rendering, and good color stability, prompting extensive research in this area.

In 2017, Qi et al. achieved excellent color stability and high efficiency in blue/orange complementary hybrid WOLEDs based on multi-EMLs. The multi-EMLs are composed of a selective insertion of an undoped orange UEML between heavily doped blue EMLs. The undoped phosphorescent UEML was composed of orange Ir complexes bis(4-tert-butyl-2-phenylbenzolatoN,C^2^’)iridium(III) (acetylacetonate)[(tbt)_2_Ir(acac)]. Here, the TADF material of bis[4-(9,9-dimethyl-9,10- dihydroacridine)phenyl]sulfone (DMAC-DPS) acted as blue emitter, which shows high Φd/Φp ratio of 4.0, high photoluminescence quantum yield (PLQY) of 90% in a neat film, short excited state lifetime (≈3.0 μs in the ordered film), and wide blue emission spectrum (FWHM ≈ 80 nm) [95]. The double DPEPO possessed an ultra-high singlet and triplet exciton energy (S1 = 3.94 eV, T1 = 2.98 eV), which could confine excitons in the EML. The device achieved minimal CIE shift of (0.008, 0.003) and maximum PE of 45.8 Lm/W with maximum EQE of 15.7% and maximum EQE of more than 12% at 1000 cd/m^2^ [96].

**Figure 12:**
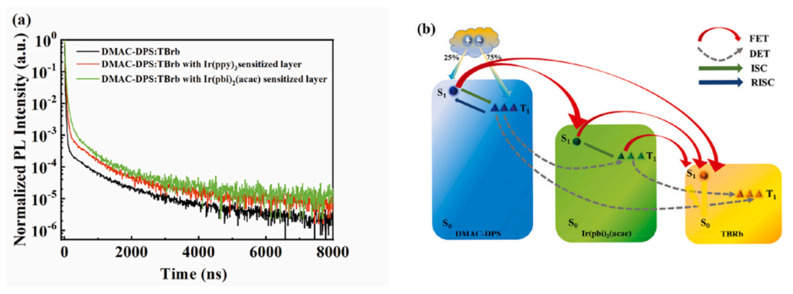
(**a**) The transient PL decayed curves of the different films observed at 570 nm; (**b**) schematic diagram of the energy transfer processes in devices [97].

**Figure 13:**
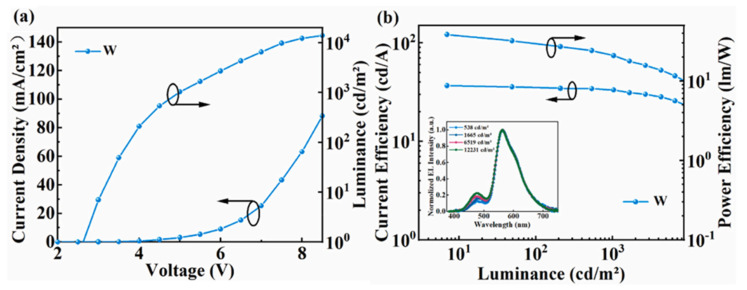
(**a**) Current–voltage–luminance curves; (**b**) current efficiency–luminance–power efficiency curves of device W; inset shows EL spectra of device at different luminances [97].

In 2023, our research group combined the undoped phosphorescent sensitization system with the TADF host of DMAC-DPS to achieve warm white devices by restraining energy transfer between the bluish-green EML and the orange UEML. Because the energy transfer from Ir(pbi)_2_(acac) to TBRb is incomplete, a thinner sensitizer was used in this white device to suppress the emission of the sensitizer, as shown in Figure 12. The maximum PE and CE of the device reached 38.5 Lm/W and 36.8 cd/A, respectively. The roll-off rate from 100 cd/m^2^ to 1000 cd/m^2^ was 7.0%, which shows high color stability, as shown in Figure 13 [97]. The improvement in performance is mainly attributed to the significant trapping effect of the ultrathin sensitization layer, which produces efficient *FRET* from the TADF host to the fluorophore [98].

In 2018, Dong et al. developed efficient and simplify WOLEDs by embedding an undoped yellow UEML within a blue EML. The EML of the WOLED utilized mCP doped with 10% 2CzPN as the blue TADF EML and undoped yellow phosphor iridium(III) bis(4-(4-tert-butylphenyl) thieno[3,2-c]pyridinato-N,C2′) acetylacetonate (PO-01-TB) as the complementary UEML embedded within the blue EML. The device achieved maximum forward view PE of 79.2 Lm/W and maximum forward view EQE of 22.3% [99]. The high performance of this device can be attributed to the exciplex formed effectively at the interface of mCP/B3PyMPM, then its energy transfers to the low-lying T1 of the blue TADF fluorophors or yellow phosphors.

In 2018, Ying et al. constructed highly efficient hybrid WOLEDs by simply inserting an undoped phosphorescent UEML into the blue emitter host of mCBP and electron acceptor of PO-T2T. Due to the energy level matching and unblocked carrier injection, the resulting WOLEDs have low operating voltage of 2.35 V. The maximum forward-looking EQE and PE reach 22.45% and 97.1 Lm/W, respectively [100]. The EL performances of doping WOLEDs based on UEMLs are summarized in Table 1.

## 4. Doping-Free WOLEDs with UEMLs

High-efficiency WOLEDs based on UEMLs are typically prepared using multiple doped EMLs. However, the performance of doped EMLs is highly sensitive to doping concentrations and depends heavily on precise doping ratios during the co-evaporation process, leading to increased complexity in fabrications, especially in WOLEDs with high CRIs, where precise controls of guest concentrations in each EML are necessary to achieve a balanced white emission with high efficiency [101]. The use of undoped UEMLs show the potential to reduce production costs due to their simple fabrication process [102]. Managing charge/exciton behavior is crucial in simplifying WOLED structures while maintaining high device performance, making it more suitable for large-scale production and industrialization [103]. The EL performances of doping-free WOLEDs based on UEMLs are summarized in Table 2.

### 4.1. Fluorescent Doping-Free WOLEDs with UEMLs

As early as 2002, Tsuji et al. described a non-doped fluorescent WOLED based on complementary colors and excitons diffusion, where the color can be adjusted by changing the position of the ultrathin exciton capture layers [104]. High CRIs are required in high-quality WOLEDs for lighting purposes [105]. In 2005, Xie et al. achieved a non-doped WOLED with high CRI through the device structure of ITO/NPB (50 nm)/TPBi (3 nm)/Alq_3_ (15 nm)/[2-methyl-6-[2-(2,3,6,7-tetrahydro-1H,5H-benzo[ij]quinolizin-9-yl)ethenyl]-4H-pyran-4-ylidene] propane-dinitrile (DCM2) (0.1 nm)/TPBi (25 nm)/Alq_3_ (10 nm)/LiF/Al. The RGB-stacked multilayer structure achieved pure white emission with CIE coordinates of (0.3198, 0.3400) under 9 V and CRI of 97 [106]. In 2013, Yang et al. proposed a new non-doped method for high CRI with double-sublayer WOLEDs using the highly blue fluorescent dye of 4,4′-Bis(2,2-diphenyl-ethen-1-yl)-4,4′-di-(tert-butyl)phenyl (p-TDPVBi) and red fluorescent dye of DCM2 together with the well-known green fluorescent dye of quinacridone (QAD). By adjusting the thickness of DCM2 and QAD, the control of the exciton RZ can be achieved, reaching maximum CE and PE of 13.54 cd/A at 12 V and 6.68 Lm/W at 5 V, respectively [107].

In 2013, Chen et al. prepared non-doped fluorescent WOLEDs with multi-UEMLs structure, with Rubrene as the yellow UEML and DPVBi as the blue emitters. DPVBi not only emits incomplete energy but also transfers the incomplete energy to Rubrene. Multi-UEMLs show excellent carrier trapping effects as the EML and capture layer. The CIE coordinates of the device at 3–7 V show minimal variation of (0.016, 0.009) [108].

**Figure 14:**
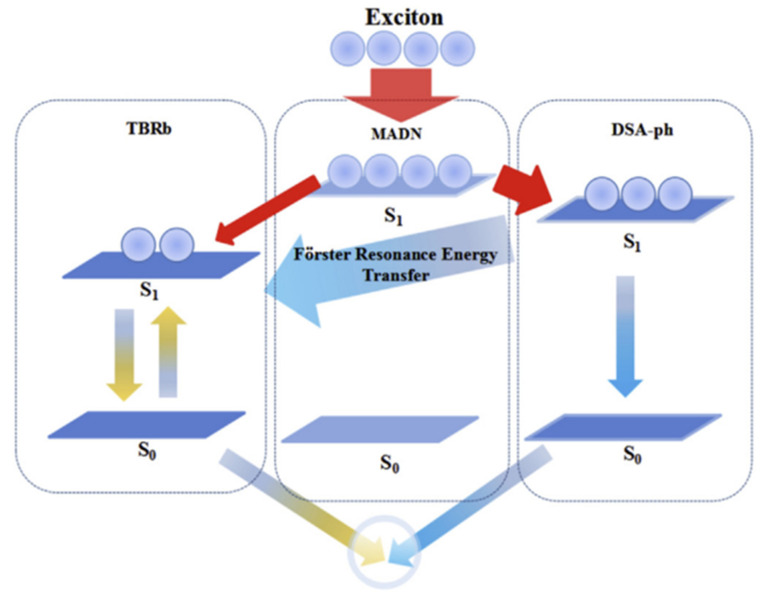
Schematic parameters and energy level diagrams of the fabricated device [109].

To meet various CCTs for lighting demands, Xu et al. demonstrated a wide color-range tunable, high-efficiency, and low-efficiency roll-off fluorescent WOLED using two complementary color undoped ultrathin emitters, a blue fluorescent UEML (DSA-ph) and a yellow fluorescent UEML (TBRb), in 2016. The IL can effectively regulate charge carriers between blue and yellow UEML and suppress the *FRET* between DSA-ph and TBRb as shown in Figure 14. The device shows maximum CE of 6.71 cd/A with operating voltage of 3.2 V [109]. By increasing the voltage, the optimal color temperature range of the designed WOLED can be tuned from warm white emission (6932 K) to cool white emission (3072 K).

### 4.2. Phosphorescent Doping-Free WOLEDs with UEMLs

The low efficiencies in fluorescent WOLEDs are critical for meeting commercial applications [110]. The combination of UEMLs and non-doped phosphorescent dye to construct the EML is an economical and effective method to achieve high-efficiency devices [111]. In 2016, Wu et al. inserted phosphorescent UEMLs with a thickness of less than 0.3 nm to prepare simple and efficient WOLEDs. The device showed maximum CE of 45.5 cd/A, PE of 46.1 Lm/W, and EQE of 17.6% [23]. The double-color device possessed CE of nearly 50 cd/A, PE of 55.5 Lm/W, and EQE of 19.3% at luminance of 1000 cd/m^2^ [23].

**Figure 15:**
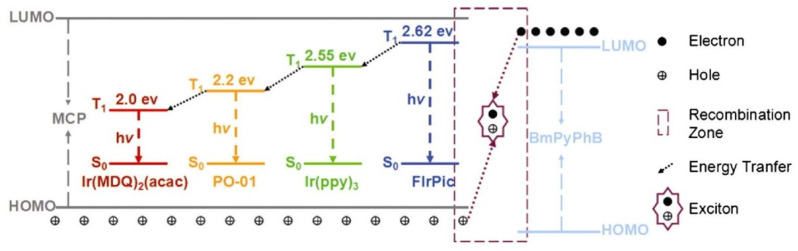
Operational principles of four colors red/orange/green/blue WOLEDs [119].

Under high current density, the lifetime and efficiency of OLEDs often accelerate degradation [112]. Tandem WOLEDs are proposed to alleviate this problem, which can obtain improvements in lifetime and brightness [113,114]. Tandem OLEDs stack multiple EL units in series through charge generation units (CGU) to achieve high efficiency and long lifetime by reducing the current density in the device [115]. In 2017, Zhang et al. used ultrathin phosphorescent dyes as the EMLs to prepare efficient non-doped tandem phosphorescent WOLEDs. The performance of the tandem electrode depends significantly on the thickness of aluminum in the CGU, which is LiF/AL/HAT-CN. By stacking two of the same white emission units, the maximum CE and EQE of the tandem white PhOLED achieved 94.9 cd/A and 31.6%, respectively [116]. The results show that UEMLs can be widely used to simplify the preparation process. For non-doped UEMLs, ILs are usually introduced to help better regulate the transport of carriers in the EML and control energy transfer [117]. Due to the large bandgap energy levels and high triplet energy, mCP is widely employed for modulating charge and exciton carriers in the IL, and researchers endeavor to develop highly efficient phosphorescent OLEDs with stable EL spectra based on this material. To simultaneously achieve high efficiency and balanced light emission, Tan et al. optimized the non-doped device structure by strategically placing three RGB UEMLs in the WOLED structure in 2015. The TAPC layers served as a hole-capturing layer, and the red dopant was a commercially available Ir-based red dye. The proximity of the TAPC layer to the blue EML resulted in efficient hole charge capture, promoting blue emission. Simultaneously, enhanced green emission was observed due to exciton diffusion. The optimized CIE coordinates (at 500 cd/m^2^) ranged from (0.441, 0.412) to (0.385, 0.428) with maximum CE and PE reaching 23.4 cd/A and 17.0 Lm/W, respectively, achieving improved exciton redistribution for more balanced light emission [118]. In 2015, our research group successfully fabricated efficient and color-stable undoped WOLEDs with multi-colors of orange/blue, red/orange/blue, and red/orange/green/blue. The mCP in the structure served as IL to control energy transfer and achieve balanced emission for white light. The designed structures suppressed the movement of charge recombination regions and direct charge-trapping effects on the UEMLs. As a result, the maximum efficiencies for two-color, three-color, and four-color WOLEDs were 30.9 cd/A (27.7 Lm/W), 30.3 cd/A (27.2 Lm/W), and 28.9 cd/A (26.0 Lm/W), respectively [119]. Such outstanding spectra and high efficiency are attributed to the stable energy transfer between EMLs, as shown in Figure 15. In 2017, Zhang et al. employed mCP as the charge/exciton modulation layer (C-EAL) to achieve highly efficient WOLEDs with double phosphorescent UEMLs. The role of mCP as C-EAL effectively suppresses carrier capture and reduces direct recombination, facilitating precise control over the distribution of carriers and excitons. Efficiency and spectra exhibit a specific dependence on the thickness of the C-EAL. When the C-EAL was approximately 2 nm, the device demonstrated maximum CE of 37.2 cd/A and PE of 36.0 Lm/W [120]. Devices based on the 4 nm C-EAL exhibit stable CIE coordinates, transitioning from (0.32, 0.40) at 124 cd/m^2^ to (0.30, 0.40) at 9705 cd/m^2^ [120].

**Figure 16:**
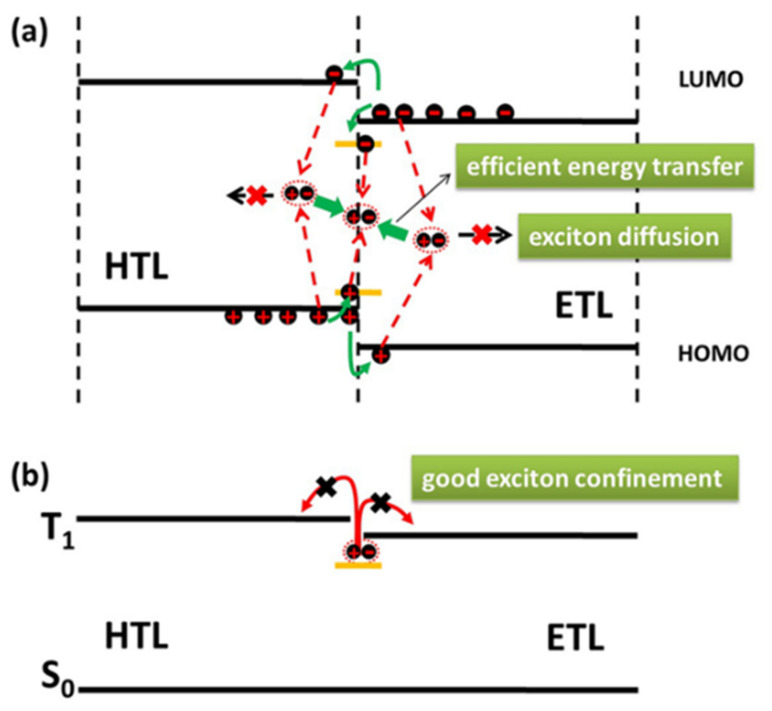
(**a**) Exciton generation, diffusion, and transfer processes at the vicinity of the undoped UEML; (**b**) exciton confinement effect of the transporting layers [121].

TCTA also usually acts as an IL to manage the formation of excitons. By precisely adjusting the positions of the IL and UEML, the probability of exciton recombination is enhanced, concurrently minimizing exciton annihilation. In 2013, Zhao et al. demonstrated WOLED structures based on UEMLs without doping technology. The introduction of UEMLs comprised of pure phosphorescent dye between HTL and ETL results in undoped PhOLEDs, which simplifies the manufacturing process. A thin TCTA IL (1 nm) was inserted between non-doped EMLs to suppress energy transfer among different dyes, achieving balanced white emission, as shown in Figure 16. Additionally, the device color could be easily adjusted by varying the thickness of the TCTA layer. The maximum EQE, CE, and PE for the RGB device were 18.5%, 34.6 cd/A, and 40 Lm/W, respectively [121]. In 2017, Zhang et al. successfully prepared high-quality warm white phosphorescent OLEDs for excellent illumination by strategically controlling the positions of red, green, and blue UEMLs. Due to the significantly lower triplet energy level of Ir(MDQ)_2_(acac) (T1 = 2.0 eV) compared to FIrpic (T1 = 2.62 eV), a 1 nm TCTA IL was introduced between the two dyes to ensure an appropriate blue light emission ratio. Moreover, because the energy transfer efficiency from BmPyPB to FIrpic is suboptimal, a 2 nm 26DczPPy (T1 = 2.71 eV) was introduced to facilitate effective energy transfer from the triplet state of 26DczPPy to FIrpic. Through meticulous control of the IL and UEML positions, the likelihood of exciton recombination is significantly enhanced, while exciton annihilation is notably suppressed. The resulting WOLEDs achieved maximum EQE of 20.3%. Additionally, within the brightness range of 1000 to 30,000 cd/m^2^, the device exhibited outstanding warm white emission with minimal variation in CIE coordinates from (0.47, 0.43) to (0.43, 0.44) with high CRI of 80 [122].

The precise control of doping concentration and co-deposition rates to form exciplex can lead to the complexity of the production process [123]. Therefore, the formation of a bilayer interface exciplex at the interface of the donor and acceptor layers is a promising approach that separates the exciton recombination and emission sites, enhancing the energy transfer pathway towards the emitter while still allowing the full utilization of triplet excitons, achieving high-performance, low-cost WOLEDs [65,124]. This approach also simplifies the device structure. In 2020, Ying et al. effectively utilized excitons by combining phosphorescent multi-UEMLs with an interface exciplex to develop high-performance, non-doped WOLEDs. mCBP was the donor component, and PO-T2T was the acceptor component of the exciplex. In this device, excitons transferred from the exciplex to the blue UEML through DET and *FRET* processes, resulting in efficient blue emission. The dual orange UEMLs were located 2 nm away from the blue UEML. The orange emission originated from four parallel multi-*FRET*s from the interface exciplex or blue UEML to the dual orange UEMLs. The resulting warm WOLED achieved forward PE and EQE of 91.5 Lm/W and 21.3% with low turn-on voltage of 2.25 V. Importantly, even at a high brightness of 1000 cd/m^2^, the PE remained 49.5 Lm/W [125].

### 4.3. Hybrid Doping-Free WOLEDs with UEMLs

**Figure 17:**
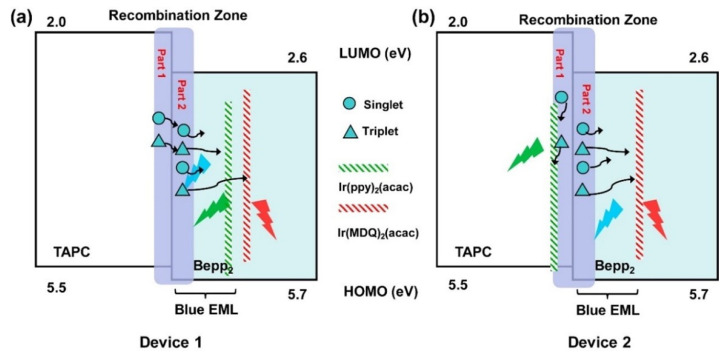
Proposed operational mechanisms for (**a**) green UEML between Bepp2 (Device 1); (**b**) green UEML between TAPC (Device 2) [127].

Fluorescent materials show superior carrier transport properties compared with phosphorescent materials. Thus, fluorescent dyes can act as EMLs by employing doping-free technology, which can combine with UEMLs to achieve WOLED [10,126]. In 2015, Zhao et al. first reported preparing three-color hybrid WOLEDs using an undoped process. The role of the TAPC as IL is to prevent the excitons of Bepp_2_ from being entirely consumed by the green dopant, as depicted in Figure 17. Because the ultrathin green phosphorescent dye can directly capture carriers, the singlet and triplet excitons in Bepp_2_ can be utilized for the blue fluorescence and red phosphorescence EMLs, resulting in more efficient and stable device performance. The device exhibited CE and PE of 23.2 cd/A, 20.5 Lm/W, respectively [127]. In 2016, Liu et al. first prepared a sun-like emission doping-free WOLED. HAT-CN was selected as the HIL due to its strong hole injection ability and combined NPB with TAPC to confine the excitons and electrons. The maximum total PE of the device was 14.6 Lm/W, and remained 12.2 Lm/W and 7.1 Lm/W at 100 and 1000 cd/m^2^, respectively. The device also showed a wide CCT range of 2325 K-8011 K and high CRI of 91.3, which is the first three-color DF-WOLED [128].

By combining the exciplex/electroplex system and UEMLs, a strategy to achieve broad EL spectra for white light emission was proposed in 2017 by Luo et al. In the structure, TAPC and TmPyPB serve as the donor and acceptor in the exciplex/electroplex system, exhibiting broad blue fluorescence emission with two emission peaks of 425 nm and 468 nm from exciplex and electroplex. By inserting red phosphorescent UEML of Ir(piq)_3_ and yellow phosphorescent UEML of Ir(dmppy)_2_(dpp) on both sides of the exciplex/electroplex system, respectively, the device achieves high-performance WOLED without blue emitter, showing EQE and CRI of 15.1% and 92.1, respectively, which are comparable to the best-doped WOLEDs with high CRIs [33].

**Figure 18:**
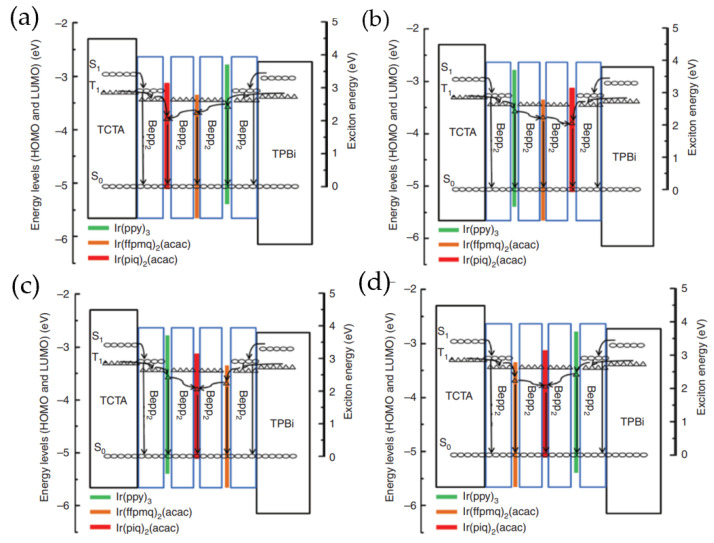
Schematic diagrams of the emission mechanisms of four white devices: **(a**) red/yellow/green; (**b**) green/yellow/red; (**c**) green/ red/yellow; (**d**) yellow/red/green [129].

**Figure 19:**
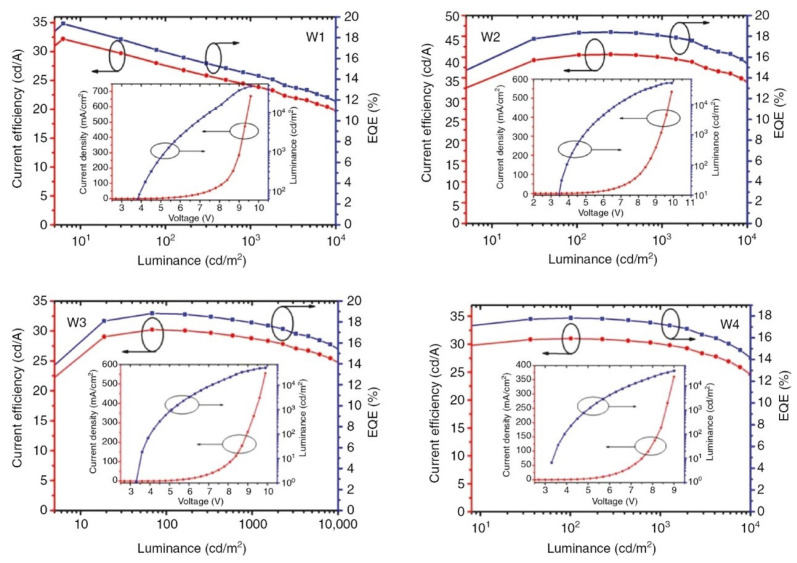
CE–L–EQE characteristic curves for four WOLEDs, Inset: J-V-L characteristic curve for the corresponding WOLED [129].

In 2017, Miao et al. introduced green, yellow, and red phosphorescent UEMLs with different arrangements into Bepp_2_ to obtain simplified hybrid WOLEDs, as shown in Figure 18. The 2 nm thick of Bepp_2_ layers between three phosphorescent UEMLs prevent energy transfer from high-level triplet excitons of TCTA and TPBi to low-level triplet excitons of phosphorescent UEMLs, balancing emission intensity across different colors. This hybrid WOLED with red/yellow/green phosphorescent UEMLs sequence exhibits remarkably high CRI of 96 and high EQE of 19.34%, achieving excellent warm white light emission with enhanced color stability, as shown in Figure 19 [129].

For ultrathin undoped hybrid WOLEDs, the positions and quantities of the ultrathin emitters have certain impacts on device efficiencies and spectra [10]. In 2018, Zhao et al. utilized multiple red phosphorescent ultrathin emitters of iridium(III) bis(2-phenylquinolinyl)acetylacetonate (Ir(pq)_2_acac) and blue fluorescent emitter bis(3-(9,9-dimethyl-9,10-dihydroacridine)phenyl)sulfone (mSOAD) to construct non-doped WOLEDs. The WOLEDs were optimized through strategic adjustment of the positions and quantities of ultrathin emitters, resulting in efficient energy transfer from mSOAD to Ir(pq)_2_acac. As a result, the device achieved outstanding performance with CE, PE, and EQE of 31.9 cd/A, 30.4 Lm/W, and 17.3%, respectively [130].

**Figure 20:**
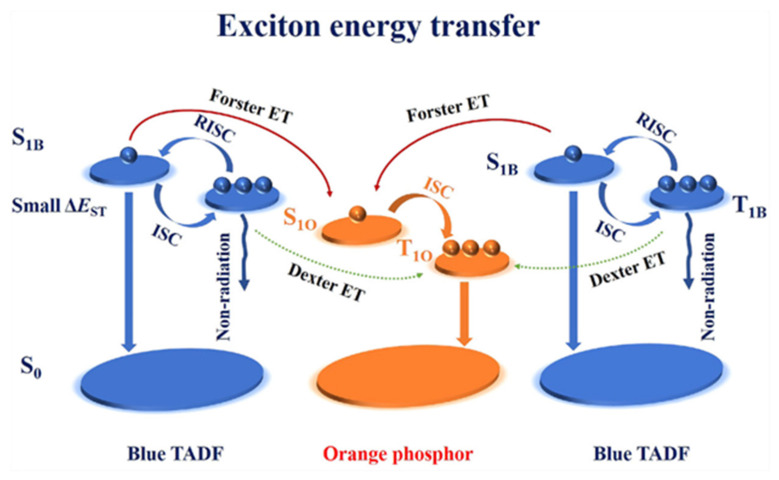
Exciton energy transfer scheme of the WOLEDs [132].

**Figure 21:**
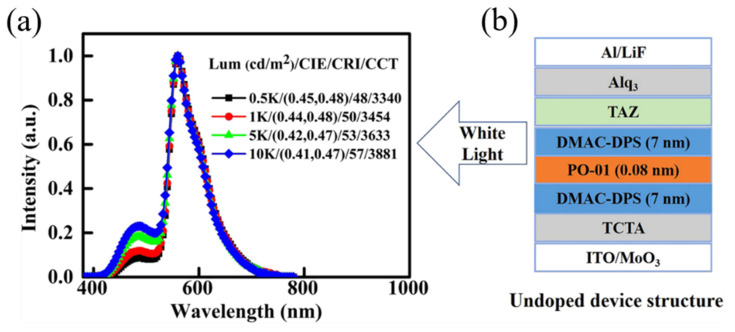
(**a**) EL spectra and corresponding CIE, CRI, and CCT values at different brightness levels of device; (**b**) device architecture [132].

In 2016, Zhao et al. utilized blue TADF emitters of DMAC-DPS and ultrathin emitter of (tbt)_2_Ir(acac) to prepare non-doped EMLs for hybrid WOLEDs. DMAC-DPS possesses a chemical structure with electron-donating and electron-accepting moieties, imparting bipolar charge transfer characteristics. By optimizing the thickness of DMAC-DPS, the WOLED achieved a maximum CE and PE of 34.9 cd/A and 29.2 Lm/W, respectively [131]. In 2020, Xue et al. employed a B-O-B emitter layer structure to achieve efficient, low-cost, and simple hybrid warm WOLEDs. The TADF DMAC-DPS effectively overcomes the drawbacks of short lifespan and poor stability associated with traditional blue phosphorescent light sources, as shown in Figure 20. The optimized WOLED demonstrates maximum EQE, PE, and CE of 9.1%, 22.7 Lm/W, and 29.0 cd/A, respectively. As the brightness increases from 1000 cd/m² to 10000 cd/m², the CIE coordinates shift from (0.44, 0.48) to (0.41, 0.47), and the CCT changes from 3454 K to 3881 K, falling within the warm white emission region, as depicted in Figure 21 [132].

**Figure 22:**
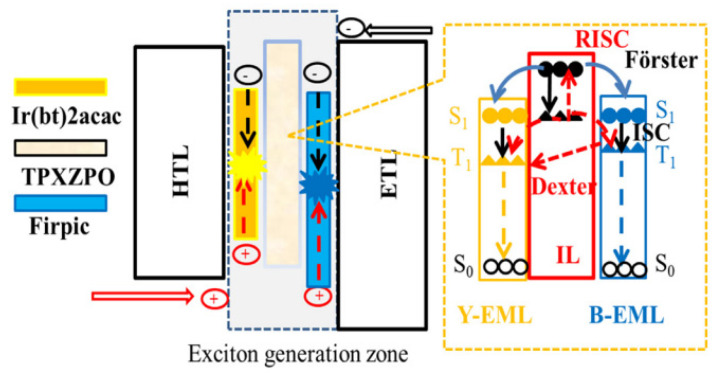
The schematic diagrams and energy transfer process of devices [136].

The TADF IL can suppress TTA effects and enhance exciton utilization efficiency [133]. Selecting an appropriate IL is crucial in regulating charges and excitons distribution, effectively controlling the emission zone and triplet excitons density [134]. In 2019, Liao et al., for the first time, chose the blue TADF material of 10,10,10-(4,4,4-phosphine-trioxotriphenylamine) trioxotriphenylamine (10Hphenoxazine) (TPXZPO) as a n-type IL switch. As the energy level of TPXZPO is higher than that of Ir(bt)_2_acac and FIrpic, TPXZPO can effectively manage excitons and charges in WOLEDs through sufficient energy transfer, as shown in Figure 22, and the generated singlets and triplets in TPXZPO might transport easily to Ir(bt)_2_acac and FIrpic by *FRET* and DET [82,135]. The device exhibited EQE of 14.96%, maximum CE of 38.93 cd/A, and maximum PE of 37.05 Lm/W with very low turn-on voltage and stable EL spectrum. In the brightness range of 100 to 10,000 cd/m^2^, the CIE coordinates only experienced a change of (0.01, 0.01) [136].

**Figure 23:**
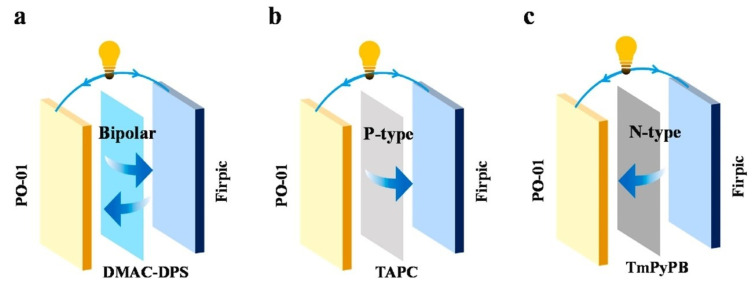
(**a**) Exciton transmission mechanism of the DMAC-DPS interlayer; (**b**) exciton transmission mechanism of the TAPC interlayer; (**c**) exciton transmission mechanism of the TmPyPB interlayer [137].

**Figure 24:**
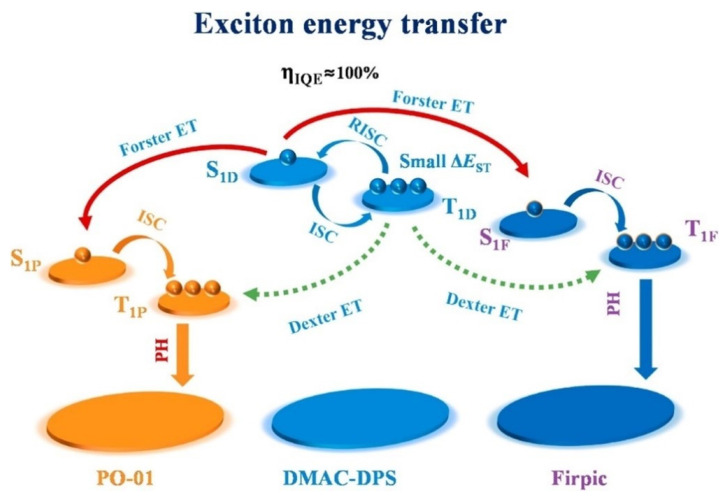
Exciton energy transfer of non-doped WOLEDs with the DMAC-DPS [137].

In 2020, Xue et al. proposed a new strategy by employing the bipolar transport TADF material of DMAC-DPS as an IL to modulate exciton distribution, as shown in Figure 23. As shown in Figure 24, due to the higher S1 and T1 energy levels of DMAC-DPS compared to PO-01 and FIrpic, the triplet excitons on DMAC-DPS can effectively upconvert to the S1 state through the *RISC* mechanism during the energy transfer process from FIrpic to PO-01 [86]. The optimized WOLED achieved maximum EQE of 15.6%, with CE and PE of 46.6 cd/A and 41.8 Lm/W, respectively [137].

**Table 2:** Summary of EL performance of doping-free WOLEDs based on UEMLs.

Devices	CIE (x, y)	CRI	Turn-On Voltage (V)	EQE_max_/PE_max_/CE_max_
				%/Lm W^−1^/cd A^−1^
Ref. [106]	(0.3198, 0.340) ^a^	97	--	-/-/-
Ref. [107]	(0.3626, 0.388) ^b^	84		-/6.68/13.54
Ref. [108]	(0.331, 0.332) ^c^	--	3	-/-/-
Ref. [109]	(0.338, 0.378) ^d^		3.2	2.126/-/6.71
Ref. [23]	(0.39, 0.43) ^e^	81	2.5	24.2/46.4/51.7
Ref. [23]	(0.38, 0.46) ^f^	-	3.2	17.6/45.5/46.1
Ref. [116]	(0.32, 0.38) ^e^	-	6	31.6/42.6/94.9
Ref. [118]	(0.419, 0.421) ^g^	74.9	-	-/17.0/23.4
Ref. [119]	(0.41, 0.43) ^e^	-	-	-/27.2/30.3
Ref. [119]	(0.401, 0.44) ^h^	-	-	-/27.7/30.9
Ref. [119]	(0.361, 0.466) ^c^	-	-	-/26.0/28.9
Ref. [120]	(0.48, 0.47) ^e^	-	-	15.0/36.0/37.2
Ref. [121]	(0.40, 0.39) ^e^	70	2.7	18.5/40.0/34.6
Ref. [122]	(0.47, 0.43) ^e^	80	2.9	20.3/39/44.2
Ref. [125]	(0.466, 0.503) ^e^	-	2.25	21.3/91.5/70
Ref. [127]	(0.44, 0.43) ^e^	82	-	-/20.5/23.2
Ref. [128]	(0.49, 0.41) ^e^	91.3	-	14.6/-/-
Ref. [33]	(0.48, 0.40) ^e^	92.1	2.85	15.1/28.2/26.9
Ref. [129]	(0.482, 0.430) ^e^	96	3.3	19.34/30.65/32.19
Ref. [130]	(0.469, 0.382) ^i^	73	2.7~3.3	17.3/30.4/31.9
Ref. [131]	(0.40, 0.46) ^c^	-	2.3	11.4/29.2/34.9
Ref. [132]	(0.44, 0.48) ^e^	50	3.1	9.1/22.7/29.0
Ref. [136]	(0.52, 0.46) ^e^	60	2.1	14.96/37.05/38.93
Ref. [137]	(0.42, 0.46) ^e^	-	2.85	15.6/41.8/46.6

EQE_max_: Maximum external quantum efficiency. PE_max_: Maximum power efficiency. CE_max_: Maximum current efficiency. ^a^ Measured at 9 V. ^b^ Measured at 6 V. ^c^ Measured at 5 V. ^d^ Measured at 3.8 V. ^e^ Measured at 1000 cd/m^2^. ^f^ Measured at 2.5mA/cm^2^. ^g^ Measured at 500 cd/m^2^. ^h^ Measured at 3000 cd/m^2^. ^i^ Measured at 7 V.

## 5. Conclusions

This article delineates the fundamental structure and concepts of WOLEDs based on UEMLs. UEMLs, an extraordinary form of dopant emitter devoid of intricate co-doping processes, exhibit distinctive advantages by simplifying device structures and reducing costs. In recent years, leveraging the merits of UEMLs, various dyes of UEML have been actively researched and applied in WOLEDs, demonstrating performance comparable to those of doping devices, thereby affirming the efficacy of UEMLs. Up to now, compared to the all-UEML structure, the strategic insertion of UEMLs with complementary color at suitable positions within the blue emission zone has proven to be an exceptionally effective method for realizing WOLEDs. However, there are few reports on the research of high-performance WOLEDs based on blue UEMLs, due lack of highly efficient blue materials. Looking ahead, there is an urgent necessity to develop stable blue emission materials exhibiting high PLQYs, T1 values, and excellent stabilities. Simultaneously, further exploration of the potential luminescent mechanisms between UEMLs and blue-emitting groups is essential to achieving even higher efficiency in WOLEDs.

## Figures and Tables

**Table 1:** Summary of EL performance of doping WOLEDs based on UEMLs.

Devices	CIE (x, y)	CRI	Turn-On Voltage (V)	EQE_max_/PE_max_/CE_max_
				%/Lm W^−1^/cd A^−1^
Ref. [38]	(0.345, 0.416) ^a^	-	~3	-/5.5/8.69
Ref. [41]	(0.34, 0.34) ^a^	72	4.5	1.2%/2.3/-
Ref. [45]	(0.45, 0.45) ^a^	87	-	15.5%/-/-
Ref. [49]	(0.35, 0.42) ^a^	65	2.7	22.2/53.4/57.1
Ref. [50]	(0.458, 0.448) ^a^	-	3.91	18.5/34.8/-
Ref. [55]	(0.32, 0.39) ^a^	55	2.71	-/39.8/40.8
Ref. [58]	(0.382, 0.437) ^a^	-	2.69	-/32.2/33.8
Ref. [63]	(0.42, 0.51) ^a^	-	2.4	20/75.3/64.5
Ref. [34]	(0.25, 0.39) ^b^	-	2.6	28.37/87.17/72.17
Ref. [34]	(0.33, 0.44) ^b^	-	2.6	23.8/81.1/67.7
Ref. [71]	(0.50, 0.43) ^a^	80	-	-/22.9/29.2
Ref. [72]	(0.36, 0.44) ^d^	85	-	-/33.42/31.91
Ref. [75]	(0.40, 0.45) ^a^	-	3.03	-/40.1/38.3
Ref. [77]	(0.329, 0.479) ^c^	54.2	2.74	15.4/48.8/47.8
Ref. [78]	(0.397, 0.523) ^a^	49	2.74	17.7/60/60.2
Ref. [79]	(0.496, 0.423) ^e^	85	2.4	21.1/46.0/36.4
Ref. [79]	(0.459, 0.426) ^a^	81	-	26.1/50/40
Ref. [87]	(0.301, 0.311) ^f^	75	2.7	-/8.9/7.6
Ref. [90]	(0.436, 0.467) ^d^	-	3.0	15.3/34.9/38.8
Ref. [92]	(0.33, 0.33) ^a^	52	2.2	12.4/34.1/32.6
Ref. [22]	(0.384, 0.459) ^f^	-	2.7	-/60.9/52.4
Ref. [96]	(0.262, 0.355) ^a^	-	3.1	15.7/45.8/45.2
Ref. [97]	(0.459, 0.483) ^g^	-	2.6	-/38.5/36.8
Ref. [99]	(0.44, 0.51) ^a^	-	2.6	22.3/79.2-
Ref. [100]	(0.420, 0.497) ^h^	-	2.35	22.45/97.1/74.2

EQE_max_: Maximum external quantum efficiency. PE_max_: Maximum power efficiency. CE_max_: Maximum current efficiency. ^a^ Measured at 1000 cd/m^2^. ^b^ Measured at 2000 cd/m^2^. ^c^ Measured at 5 V. ^d^ Measured at 6 V. ^e^ Measured at 9 V. ^f^ Measured at 207 cd/m^2^. ^g^ Measured at 538 cd/m^2^. ^h^ Measured at 4 V.

## Data Availability

Not applicable.

## Abbreviations

Abbreviations and full spelling of terms mentioned in the article.

**Abbreviation**	**Full Name**
OLEDs	organic light-emitting diodes
WOLEDs	white organic light-emitting diodes
EML	emitting layer
UEML	ultrathin emitting layer
EQE	external quantum efficiency
TTA	triplet–triplet annihilation
AFM	atomic force microscopy
S1	singlet state
IQE	internal quantum efficiency
T1	triplet excitons
DET	Dexter energy transfer
*FRET*	Förster energy transfer
TADF	thermally activated delayed fluorescence
*RISC*	reverse intersystem crossing
$\Delta E_{ST}$	energy gap
FWHM	full width at half maxima
EL	electroluminescence
DSA-ph	P-bis(p-N, N-diphenylaminostyryl)benzene
MADN	2-methyl-9,10-di(2-naphthyl)anthracene
Rubrene	5,6,11,12-tetraphenyl-naphthacene
PE	power efficiency
UPL	ultrathin premixed layer
fvin	4-di(4′-tert-butylbiphenyl-4-yl)aminobiphenyl-4′-yl)acrylonitrile
fcho	4-di(4′-tert-butylbiphenyl-4-yl)aminobenzaldehyde
HTL	hole transport layer
CIE	Commission Internationale de l’Eclairage
CRI	color rendering index
IL	interlayer
ETL	electron transport layer
Ir(bt)_2_(acac)	bis(2-phenyl-benzothiozolato-N,C2’)iridium(acetylacetonate)
FIrpic	bis(3,5-difluoro-2–(2-pyridyl)phenyl-(2-carboxypyridyl))iridium III
TCTA	4,4,4″-tris(N-carbazolyl)triphenyl-amine
26DCzPPy	2,6-bis(3-(carbazol-9-yl)phenyl)-pyridine
CE	current efficiency
HTM	hole transporting material
Ir(_2_-phq)_2_(acac)	bis(2-phenylquinoline)-(acetylacetonate)-iridium(III)
Ir(ppy)_3_	fac-tris(2-phenyl-pyridinato)-iridium(III)
RZ	recombination zone
ETM	electron transport material
PO-01	Iridium(III) bis(4-phenylthieno[3,2-c]pyridinato-N, C2′) acetylacetonate
TPBi	2,2′,2″-(1,3,5-Benzinetriyl)-tris(1-phenyl-1-H-benzimidazole)
mCP	N,N′-dicarbazolyl-3,5-benzene
B3PYMPM	bis-4,6-(3,5-di-3-pyridyl-phenyl)-2-methylpyrimidine
mCBP	4,4’-bis(3-mehtylcarbazol-9-yl)-2,2’-biphenyl
PO-T2T	1,3,5-triazine-2,4,6-triyl)tris(benzene3,1-diyl))tris(diphenylphosphine oxide
CCT	correlated color temperature
EAL	excitons adjustment layer
FSF4A	N-([1,10-biphenyl]-2-yl)-N(9,9-dimethyl-9h-fluoren-2-yl)-9,90-spirobi[fluorene]-4-amine
(tbt)_2_Ir(acac)	bis(4-tert-butyl-2-phenylbenzo[4,5]thiazolo-[3,2-a]pyridin-5-yl)iridium(III) (acetylacetonate)
DMAC-DPS	bis[4-(9,9-dimethyl-9,10-dihydroacridine)phenyl]sulfone
PLQY	photoluminescence quantum yield
PO-01-TB	Iridium(III) bis(4-(4-tert-butylphenyl) thieno[3,2-c]pyridinato-N,C2′d) acetylacetonate
DCM2	[2-methyl-6-[2-(2,3,6,7-tetrahydro-1H,5H-benzo[ij]quinolizin-9-yl)ethenyl]-4H-pyran-4-ylidene] propane-dinitrile
p-TDPVBi	4,4′-Bis(2,2-diphenyl-ethen-1-yl)-4,4′-di-(tert-butyl)phenyl
QAD	quinacridone
CGU	charge generation units
C-EAL	charge/exciton modulation layer
Ir(pq)_2_acac	iridium(III) bis(2-phenylquinolinyl)acetyl-acetonate
mSOAD	bis(3-(9,9-dimethyl-9,10-dihydroacridine)phenyl)sulfone
TPXZPO	10,10,10-(4,4,4-phosphine-trioxotriphenylamine) trioxotriphenylamine (10Hphenoxazine)
